# Baicalin Alleviates LPS-Induced Oxidative Stress via NF-κB and Nrf2–HO1 Signaling Pathways in IPEC-J2 Cells

**DOI:** 10.3389/fvets.2021.808233

**Published:** 2022-01-25

**Authors:** Minglong Bao, Mei Liang, Xinyi Sun, Sahar Ghulam Mohyuddin, Shengwei Chen, Jiaying Wen, Yanhong Yong, Xingbin Ma, Zhichao Yu, Xianghong Ju, Xiaoxi Liu

**Affiliations:** Department of Veterinary Medicine, College of Coastal Agricultural Sciences, Guangdong Ocean University, Zhanjiang, China

**Keywords:** baicalin, lipopolysaccharide, inflammatory effect, oxidative stress, NF-κB, Nrf2-HO-1

## Abstract

Baicalin is a natural plant extract with anti-inflammatory and anti-oxidant activities. However, the molecular mechanism of baicalin on oxidative stress in IPEC-J2 cells exposed to LPS remains to be unclear. In this study, LPS stimulation significantly increased Toll-like receptor 4, tumor necrosis factor-α, and interleukins (IL-6 and IL-1β) expression in IPEC-J2 cells, and it activated the nuclear factor (NF-κB) expression. While, baicalin exerted anti-inflammatory effects by inhibiting NF-κB signaling pathway. LPS stimulation significantly increased the levels of the oxidative stress marker MDA, inhibited the anti-oxidant enzymes catalase and superoxide dismutase, which were all reversed by baicalin pre-treatment. It was found that baicalin treatment activated the nuclear import of nuclear factor-erythroid 2 related factor 2 (Nrf2) protein, and significantly increased the mRNA and protein expression of its downstream anti-oxidant factors such as heme oxygenase-1 and quinone oxidoreductase-1, which suggested that baicalin exerted anti-oxidant effects by activating the Nrf2-HO1 signaling pathway. Thus, pretreatment with baicalin inhibited LPS - induced oxidative stress and protected the normal physiological function of IPEC-J2 cells via NF-κB and Nrf2–HO1 signaling pathways.

## Introduction

Diarrhea is one of the most common causes of piglet weaning mortality, and it has a significant influence on the pig industry (1). The development of diarrhea in piglets is usually accompanied by intestinal inflammation and oxidative stress, which in turn induces damage in the intestinal epithelium (2, 3). Intestinal epithelial cells (IECs) are important sites of nutrient digestion and absorption in mammals, as well as important barriers for the entry of pathogenic microorganisms and toxins into the circulatory system (4). The maintenance of normal physiological function in animals requires intact and healthy intestinal epithelial tissue (5). IPEC-J2 cells, a jejunal epithelial cell line isolated from newborn piglets, represent an important model for studying porcine intestinal function *in vitro* (6). IPEC-J2 are cell line used as model in many studies. For example, IPEC-J2 cells can be used to study the interaction between E. coli and its host, the damage of mycotoxin and cadmium exposure to pig intestines, and the regulatory effect of *Lactobacillus salivarius* on pig intestines (7). The occurrence of intestinal inflammation and oxidative stress will lead to the deterioration of intestinal barrier function, thereby permitting harmful substances to enter the blood and leading to the occurrence of digestive system diseases (8–10). Even more, some pathogens just like *Samonella* can also exploit inflammation to penetrate enterocytes after the intestinal barrier was disrupted (11, 12).

The inflammatory response is a type of resistance to foreign pathogens. Inflammatory factors produced by the body during the inflammatory response disrupt the normal physiological structure of the intestinal epithelium and play a role in resisting the invasion of foreign pathogens. However, excessive inflammatory responses can damage bodily tissues, thereby suppressing intestinal barrier function (13). IECs recognize extracellular stimuli through Toll-like receptors (TLRs) on the cell surface (14), which can recognize lipopolysaccharide (LPS), a tissue component of the outer wall of gram-negative bacteria; this leads to the stimulation of nuclear factor (NF-κB) mediated pro-inflammatory cytokine production and immune cell recruitment (15). The recruited immune cells secrete pro-inflammatory factors that induce apoptosis of IECs, thereby disrupting intestinal epithelial integrity (16).

Oxidative stress is induced when free radicals in the body attack tissues and cells (17). Under normal conditions, free radicals and other oxides are in a dynamic equilibrium with anti-oxidants in the body, and oxidative stress does not occur. When the balance is disrupted by some cause (e.g., acute hypoxia, drug toxicity, acute inflammatory response), the levels of highly oxygenated reactive factors (e.g., free radicals, reactive oxygen species [ROS]) are increased, which leads to environmental damage in the organism, including DNA, mitochondrial, and organelle damage (18), Under normal circumstances, Keap1 binds with actin in the cytoplasm, and Nuclear factor-erythroid 2 related factor 2 (Nrf2) is a crucial transcription factor associated in the response to oxidative stress (19). When oxidative stress occurs, Keap1 dissociates from Nrf2, which enters the nucleus and binds to its corresponding target sites in DNA to induce the synthesis of corresponding detoxification and anti-oxidant enzymes (20). Therefore, the body's endogenous anti-oxidant signaling pathway (Keap1–Nrf2/ARE) has a significant anti-oxidant stress effect.

Baicalin (C21H18O11) is a flavonoid extracted from the natural plant Scutellaria baicalensis that has biological functions such as anti-inflammatory (21), anti-oxidant (22), and anti-viral effects (23). For example, baicalin can reverse LPS-induced inflammation in H9c2 cells (24). Baicalin exerted anti-oxidant effects against lung injury (25). Moreover, it has good therapeutic effects in various animal models of inflammation (26, 27), and oxidative stress *in vitro* (28). However, few reports were focused on the oxidative stress response of inflammation in the intestine of piglets (29). Thus, the present study observed the specific mechanism of LPS-induces oxidative stress in IPEC-J2 cells and the therapeutic effects of baicalin.

## Materials and Methods

### Construction of the Experimental Model

Porcine IECs (IPEC-J2) (The IPEC-J2 cells obtained from the Guoqiang Zhu of China Yangzhou University) were cultured in Dulbecco's modified Eagle's medium (DMEM/F-12 (1:1) basic, C11330500BT, Gibco, USA) supplemented with 10% fetal bovine serum (FBS, Z7186FBS, ZETA, France), 50 IU/mL penicillin, and 50 μg/mL streptomycin (Penicillin-Streptomycin, 15140-122, Gibco) at 37°C in a 5% CO2 atmosphere. The medium was replaced 48 h after the initial cell culture. When cells reached 70% confluence, they were stimulated with 40 μg/mL LPS from Escherichia coli (L2880, Sigma, USA) for 1 h or 10 mol/mL H_2_O_2_ for 3 h after pretreatment with baicalin for 24 h.

Baicalin (C_21_H_18_O_11_, purity 95.4%, Jiu- Ling- Cao biotechnology Company, Wuhan, China), which can be found in the pharmacopeia of China, were stored at the room temperature. Firstly, baicalin was dissolved in the DMSO as mother liquor (400 μg/mL). Secondly, an appropriate amount of mother liquor was added to the cell culture medium. In this way, baicalin solution with the required concentration was prepared. To assess viability, equal numbers of IPEC-J2 cells were seeded in 96-well plates (Wuxi NEST Life Technology Co., Ltd) and DMEM/F12 medium with 10% FBS was used to grow the cells. After achieving 95% viability, the cells were rinsed two times with PBS, serum starved for 2 h, and treated with various doses of baicalin (4–64 μg/mL) for 24 h, followed by the addition of 10 μl of CCK-8 reagent (C0038, Dojindo, Japan) to each well. Cells were incubated at 37°C in 5% CO2 for 1 h before absorbance was measured at 450 nm using a microplate reader.

To determine cell viability after baicalin treatment, equal numbers of IPEC-J2 cells were seeded in 96-well plates and cultured in DMEM/F12 medium containing 10% FBS. After getting 95% confluence, the cells were rinsed two times with PBS, serum starved for 2 h, and treated with different concentrations of baicalin for 24 h (4–16 μg/mL). The cells were then serum-starved for 2 h and treated with 40 μg/mL LPS for 1 h before adding 10 μl of CCK-8 reagent to each well. IPEC-J2 cells were incubated at 37°C with 5% CO2 for 1 h before absorbance was measured at 450 nm using a microplate reader.

### ELISA

After IPEC-J2 cells were cultured to 95% confluence in six-well plates (Wuxi NEST Life Technology Co., Ltd), they were rinsed two times with PBS, serum starved for 2 h, and treated with different concentrations of baicalin for 24 h (4–16 μg/mL). Cells were then washed twice with PBS and serum-starved for 2 h, and supernatant was collected after treatment with 40 μg/mL LPS for 1 h. IL-6 (MM-0418O2, Meimian, China), IL-1β (MM-0422O2, Meimian), TNF-α (MM-0383O2, Meimian), MDA (MM-191601, Meimian), SOD (MM-0450O2, Meimian), and CAT levels (MM-32849O2, Meimian) were examined via ELISA according to the manufacturer's instructions. Normalization was performed according to the concentration of cellular proteins. The experimental groups and drug concentrations used are presented in Table 1.

**Table 1:** Experimental groups and drug concentrations.

**Groups**	**Drug administration**
Control group	IPEC-J2 (not treated)
Lipopolysaccharide (LPS)	IPEC-J2 (40μg/mL LPS)
H_2_O_2_	IPEC-J2 (H_2_O_2_ 10mol/mL)
Low baicalin	IPEC-J2 (40μg/mL LPS)+4μg/mL Baicalin
Medium baicalin	IPEC-J2 (40μg/mL LPS)+8μg/mL Baicalin
High baicalin	IPEC-J2 (40μg/mL LPS)+16μg/mL Baicalin

### RNA Extraction, Reverse Transcription, and Real-Time PCR

RNA was extracted from IPEC-J2 cells using TRIzol reagent (Invitrogen, Waltham, MA, USA) according to the manufacturer's instructions, and RNA concentrations were determined using an OD1000 instrument. The isolated RNA was used to reverse-transcribe cDNA using EasyScript One-Step gDNA Removal and cDNA Synthesis SuperMix (AE311, EasyScript, China) according to the manufacturer's instructions, cDNA was amplified using EasyScript green qPCR SuperMix EasyScript Green qPCR SuperMix (AQ101, EasyScript) and specific primers. Reverse transcription-generated cDNA encoding β-actin, IL-6, IL-1β, IL-8 TNF-α, TLR4, HO-1, and NQO-1 was amplified by real-time PCR using selective primers (Table 2).

**Table 2:** Primers used for real-time PCR.

**Gene**	**Primer sequence(5**^′^-3^′^**)(30, 31)**	**Gene location**
β-actin	F: GCTGTCCCTGTATGCCTCT	NC_010445.4
	R: GATGTCACGCACGATTTCC	
IL-6	F: ATAAGGGAAATGTCGAGGCTGTGC	NC_010451.4
	R: GGGTGGTGGCTTTGTCTGGATTC	
IL-1β	F: CAAGCCAGAGAAGCAAGGTGTCC	NC_010445.4
	R: GCCGTCCTCAGCAGCAAGAAG	
TNF-α	F: AAAGGACACCATGAGCACGGAAAG	NC_010449.5
	R: CGCCACGAGCAGGAATGAGAAG	
TLR4	F: GCCATCGCTGCTAACATCATC	NC_010443.5
	R: CTCATACTCAAAGATACACCATCGG	
HO-1	F: TGCTGAATGCCTGAATGCCTGTC	NW_018084968.1
	R: TCCTGCCTCCCTCAACTGTGTG	
NQO-1	F: TGTAGACCGGGTTCTCCTTG	NC_010448.4
	R: TGAATTACATCTCTGTGGTTTA	
IL-8	F:CTCCGTGGCTCCCAAGAATTTCTC	NC_010450.4
	R:GACCAGCACAGGAATGAGGCATAG	

### Western Blotting

Proteins were extracted from IPEC-J2 cells using RIPA lysis buffer (P0013B, Beyotime, China) and a Nuclear and Cytoplasmic Protein Extraction Kit (KGP1000, KeyGEN, China). Proteins were quantified using a BCA protein assay kit (KGP1100, KeyGEN). Proteins (20 μg/sample) were separated by SDS-PAGE (Tricine-SDS-PAGE Gel Kit, CW2384S, Cwbio, China), transferred to nitrocellulose membranes (88585, Pierce, Rockford, USA), and then hybridized with specific antibodies. The following antibodies were used: NF-κB p65 (#8242, Cell Signaling Technology, Danvers, MA, USA), phospho–NF-κB p65 (#3033, Cell Signaling Technology), IκBα (#4814, Cell Signaling Technology), phospho-IκBα (#2859, Cell Signaling Technology), Nrf2 (#12721, Cell Signaling Technology), anti–HO-1 (EP1391Y, Abcam, UK), lamin B1 (ab16048, Abcam, UK), and β-actin (CW0096, Cwbio). Total protein and cytoplasmic protein levels were normalized using β-actin expression to correct for differences in protein loading. Nuclear protein blots were normalized using lamin A/C to correct for differences in protein loading. Blots were visualized using an ECL detection system, and proteins were quantified using a ChemiDoc XRS+ image analyzer (Bio-Rad, Hercules, CA, USA).

### Immunofluorescence

After reaching approximately 50% confluence, IPEC-J2 cells were treated with drugs, fixed using 4% paraformaldehyde on ice for 15 min, washed three times with PBS, permeabilized on ice with 0.2% Triton-X 100 for 5 min, and washed three times with PBS. Cells were then blocked with 1% fetal bovine serum for 30 min, incubated on ice with primary antibody (1:100 dilution) overnight, and washed three times with PBS. Subsequently, Biotin-SP (long spacer) AffiniPure Donkey Anti-Mouse IgG (H+L) (715-065-151, Jackson Immunoresearch, UK) diluted 1:100 was added dropwise at 37°C. After incubation for 1 h, cells were washed three times with PBS, and a 1:100 dilution of a594-sav (016-580-084, Jackson Immunoresearch, UK) was added dropwise at 37°C. After incubation for 1 h, cells were washed three times with PBS. DAPI (1:500 dilution) was added dropwise for 3 min at room temperature. Images of IPEC-J2 cells were acquired using a confocal microscope (Nikon, Tokyo, Japan). Antibodies against phospho–NF-κB p65 and Nrf2 were used.

### Data Analysis

When analyzing more than three means, the Student's *t*-test or one-way analysis of variance were used, and Tukey's multiple comparison test was used. The results were presented as a mean ± SEM, and *p* < 0.05 denoting statistical significance. SPSS12.0 software (IBM Company, Chicago, USA) was used for statistical analysis, and Origin 6.0 (National Institute of Health NY, USA) was used to make graphs.

## Results

### Cytotoxicity of Baicalin and Its Effects in Inflammatory and Oxidative Stress Models

**Figure 1:**
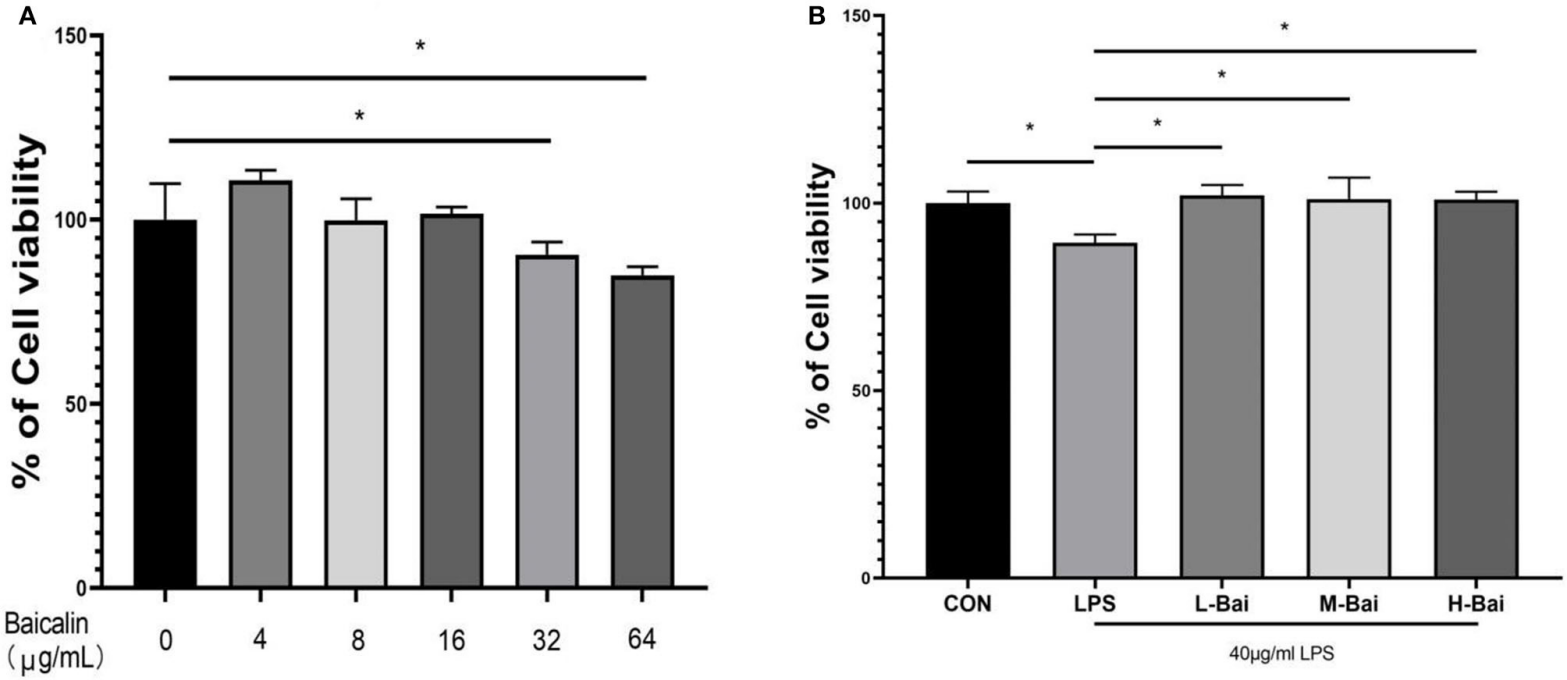
Cytotoxicity of baicalin in IPEC-J2 cells and the effect of baicalin on the viability of IPEC-J2 cells stimulated with LPS. Baicalin at 4 μg/mL increased the viability of IPEC-J2 cells, whereas 16 μg/mL baicalin was not cytotoxic to IPEC-J2 cells. Meanwhile, baicalin at concentrations of ≥ 64 μg/mL was significantly cytotoxic **(A)**. LPS stimulation decreased IPEC-J2 cell viability, and this effect alleviated by baicalin pretreatment **(B)**. Data are presented as the mean ± SEM (*n* = 6 per treatment, *p < 0.05 compared to the model group). CON, control; L-Bai, low-concentration baicalin; M-Bai, middle-concentration baicalin; H-Bai, high-concentration baicalin.

To select the appropriate concentration of baicalin for treating IPEC-J2 cells, cell viability was determined using the CCK-8 method after cells were incubated with different concentrations of baicalin for 24 h. Baicalin at 4 μg/mL (*p* < 0.05) significantly increased IPEC-J2 cell viability (Figure 1A). Baicalin treatment at 8–16 μg/mL for 24 h did not significantly alter the viability of IPEC-J2 cells (Figure 1A). IPEC-J2 cell viability was significantly inhibited following treatment with Baicalin at ≥ 32 μg/mL (*p* < 0.01) for 24 h (Figure 1A). To investigate the regulatory effect of baicalin on IPEC-J2 cells, we therefore created low- (4 μg/mL), middle- (8 μg/mL), and high-concentration groups (16 μg/mL). In an inflammation model, LPS (*p* < 0.05) significantly reduced IPEC-J2 cell viability (Figure 1B), whereas low, middle, and high concentrations of baicalin reversed this effect (*p* < 0.05, Figure 1B).

### Effect of Baicalin on Inflammatory Factors in IPEC-J2 Cells

**Figure 2:**
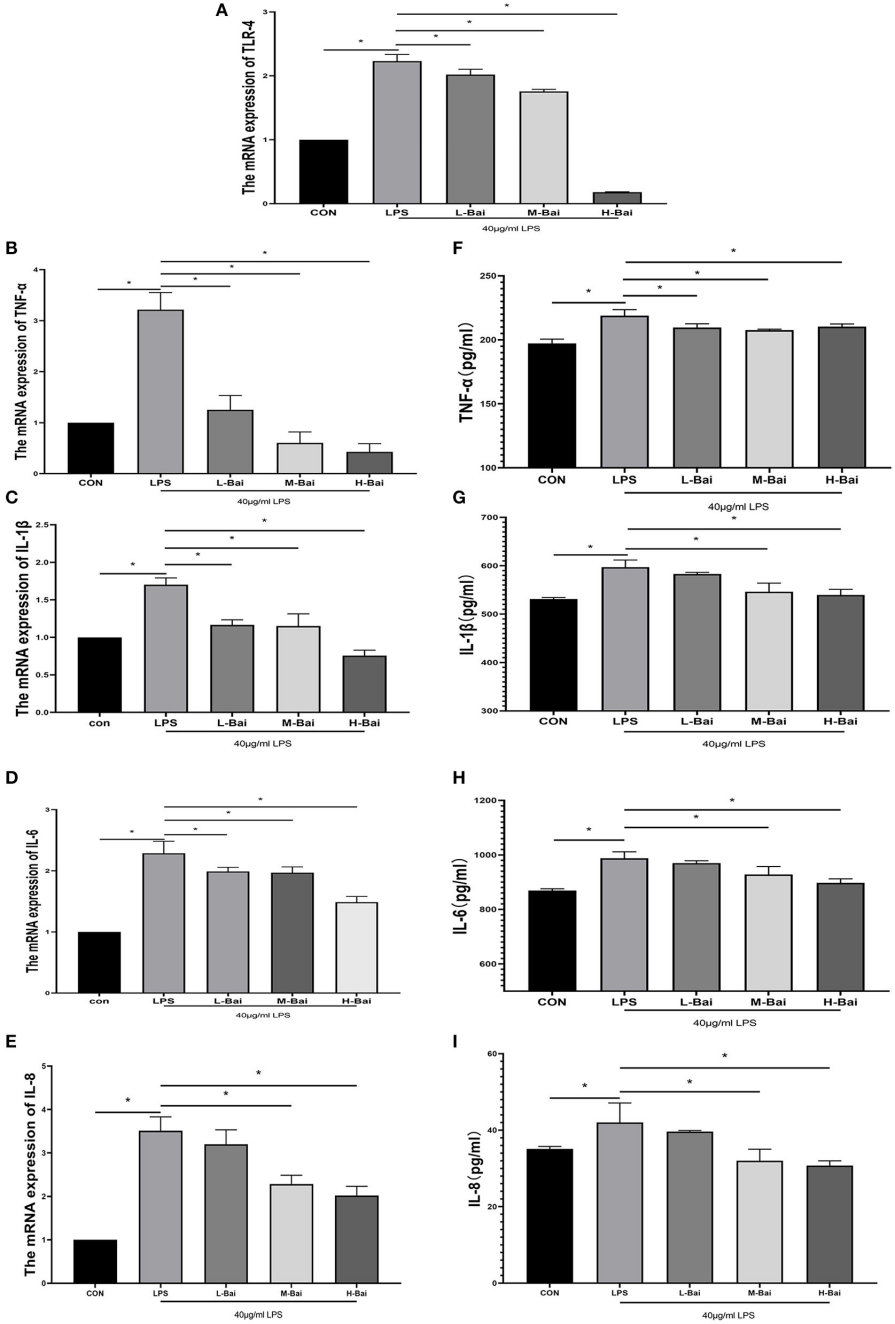
Effects of baicalin on inflammatory factor expression in LPS-stimulated IPEC-J2 cells. Quantitative effects of baicalin on TLR4 **(A)**, TNF-α **(B)**, IL-1β **(C)**, IL-6 **(D)** and IL-8 **(E)** mRNA expression in IPEC-J2 cells. Effects of baicalin on TNF-α **(F)**, IL-1β **(G)**, IL-6 **(H)** and IL-8 **(I)** protein expression in IPEC-J2 cells. Data are presented as the mean ± SEM (*n* = 3 per treatment, *p < 0.05 compared with the model group). CON, control; L-Bai, low-concentration baicalin; M-Bai, middle-concentration baicalin; H-Bai, high-concentration baicalin.

**Figure 3:**
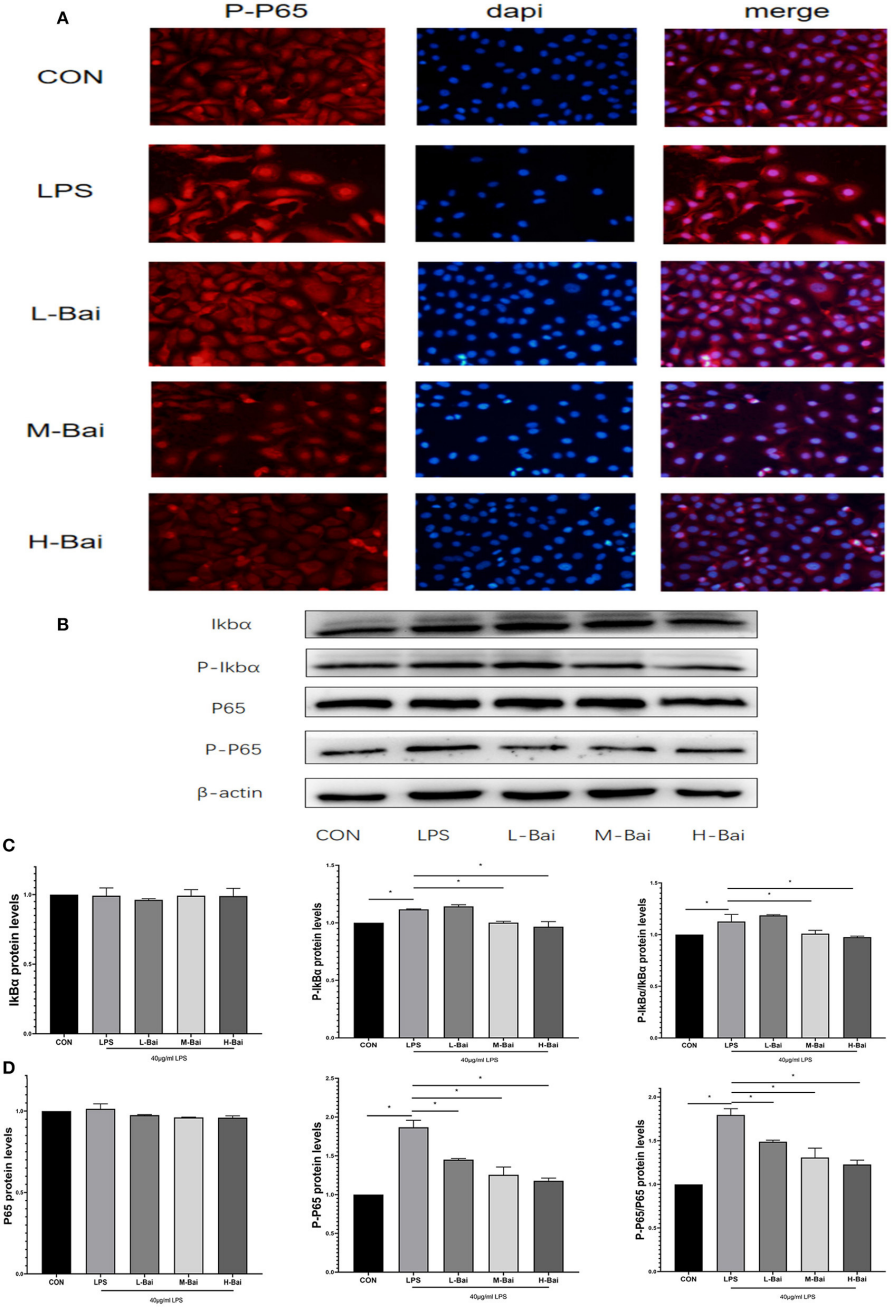
Effects of baicalin on key proteins in the NF-κB signaling pathway in LPS-stimulated IPEC-J2 cells. Immunofluorescence detection of P-p65 protein nuclear import in IPEC-J2 cells **(A)**. Western blotting was used to determine the expression of p65, P-p65, IκBα, and P-IκBα **(B)**. p65 and P-p65 protein expression and their ratio **(C)**. IκBα and P-IκBα protein expression and their ratio **(D)**. Data are presented as the mean ± SEM (*n* = 3 per treatment, *p < 0.05 compared with the model group). CON, control; L-Bai, low-concentration baicalin; M-Bai, middle-concentration baicalin; H-Bai, high-concentration baicalin.

Interleukin IL-6, IL-1β,IL-8 and tumor necrosis factor TNF-α are main cellular inflammatory factors that play significant roles in the inflammatory response of IPEC-J2 cells. According to enzyme-linked immunosorbent assay (ELISA), LPS exposure significantly increased the mRNA expression of TLR4, IL-6, IL-1β, IL-8 and TNF-α (*p* < 0.05) in IPEC-J2 cells (Figures 2A–E). Low, middle, and high concentrations of baicalin (*p* < 0.05) significantly reversed the upregulation of IL-1β in IPEC-J2 cells induced by LPS (Figure 3C). Baicalin (*p* < 0.05) concentration-dependently reduced the mRNA expression of TLR4, IL-6, IL-8 and TNF-α in LPS-stimulated IPEC-J2 cells (Figures 2A,B,D,E). Meanwhile, LPS (*p* < 0.05) stimulation significantly increased IL-6 protein expression in IPEC-J2 cells (Figure 2H). Although low-concentration baicalin did not reverse the effect of LPS on IL-6 expression, the middle and high concentrations of this drug significantly suppressed IL-6 protein expression (*p* < 0.05, Figure 2H. LPS (*p* < 0.05) stimulation significantly upregulated IL-1β and IL-8 protein expression in IPEC-J2 cells (Figures 2G,I),which was reduced by middle and high concentrations of baicalin (*p* < 0.05, Figures 2G,I). LPS (*p* < 0.05)stimulation concentrations of baicalin reverse this effect (*p* < 0.05, Figure 2F). significantly increased TNF-α protein expression in IPEC-J2 cells (Figure 2F), and all concentrations of baicalin reverse.

### Effect of Baicalin on NF-κB Expression and Signaling in IPEC-J2 Cells

The NF-κB signaling pathway is important for the inflammatory response. NF-κB signaling proteins in IPEC-J2 cells were detected by western blotting. Fluorescence microscopy of normal IPEC-J2 cells is presented in Figure 3A. LPS stimulation significantly increased the entry of P-p65 protein into the nuclei of IPEC-J2 cells. However, baicalin concentration-dependently reversed this effect (Figure 3A). LPS stimulation and baicalin treatment did not significantly affect p65 and inhibitor of κB alpha (IκBα) protein expression in IPEC-J2 cells (Figures 3C,D). LPS (*p* < 0.05) stimulation dramatically increased the protein expression of P-IκBα in IPEC-J2 cells, and this effect was canceled by the middle and high concentrations of baicalin (Figures 3B,D). The ratio of P-IκBα to IκBα displayed a similar trend as P-IκBα expression (Figure 3D). Stimulation with LPS (*p* < 0.05) significantly increased the protein expression of P-P65 in IPEC-J2 cells, whereas baicalin treatment reversed this effect in a concentration-dependent manner (*p* < 0.05, Figures 3C,D). The ratio of P-p65 to p65 exhibited a similar trend as P-p65 expression (Figure 3C).

### Effect of Baicalin on Oxidase and Malondialdehyde (MDA) Levels in an IPEC-J2 Cell Oxidation Model

**Figure 4:**
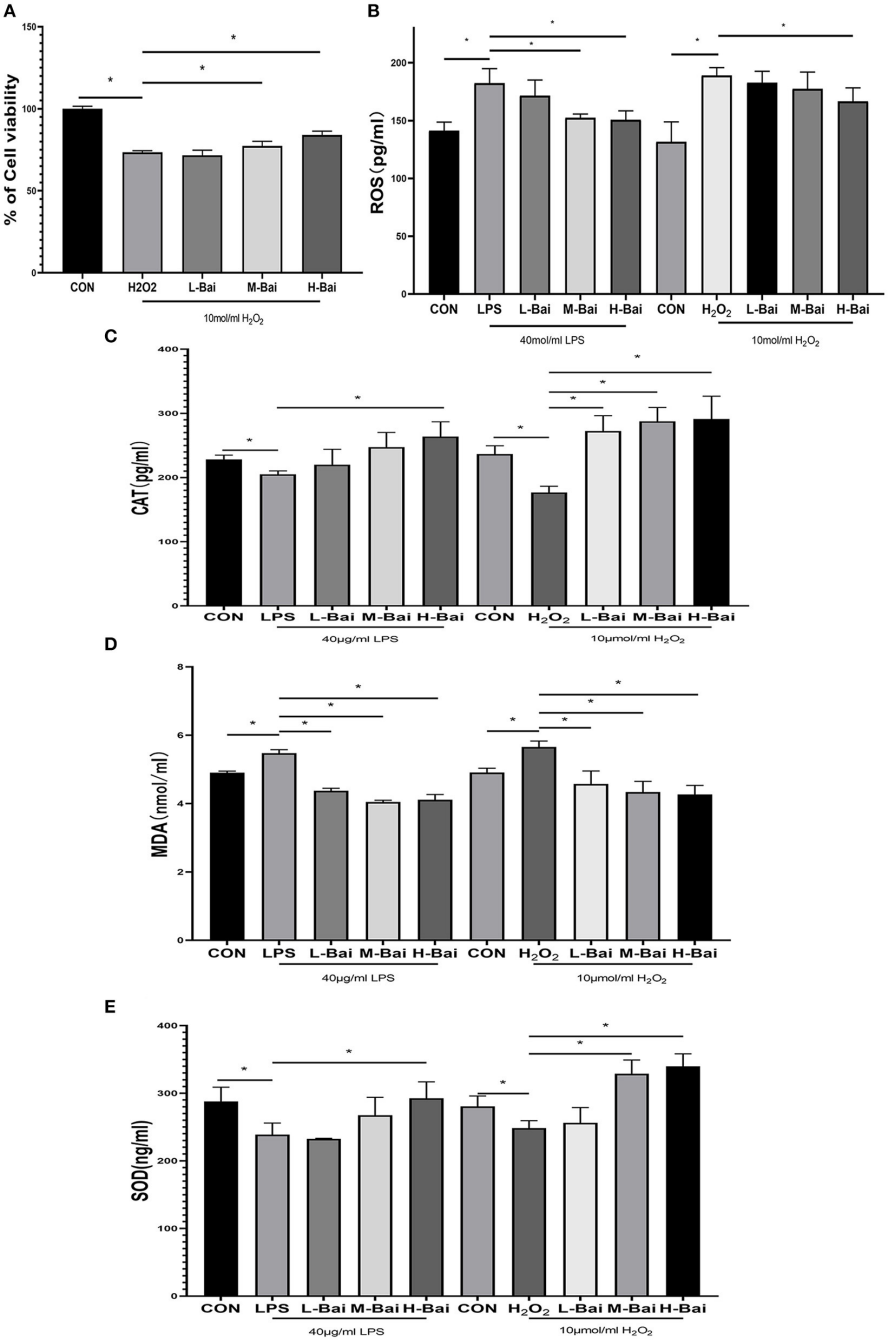
Effects of baicalin on anti-oxidant enzymes and oxidative indices in LPS-stimulated IPEC-J2 cells. H_2_O_2_ stimulation decreased the viability of IPEC-J2 cells, and this effect was alleviated by baicalin pretreatment **(A)**. Effect of baicalin ROS **(B)**, SOD **(C)**, CAT **(D)**, and MDA **(E)** levels in IPEC-J2 cells stimulated with LPS and H_2_O_2_. Data are presented as the mean ± SEM (*n* = 3 per treatment, *p < 0.05 compared with the model group). CON, control; L-Bai, low-concentration baicalin; M-Bai, middle-concentration baicalin; H-Bai, high-concentration baicalin.

In an oxidative stress model, H_2_O_2_ (*p* < 0.05) significantly inhibited the viability of IPEC-J2 cells (Figure 4A), and this effect was only reversed by high-concentration baicalin (*p* < 0.05, Figure 4A).

Superoxide dismutase (SOD), catalase (CAT), MDA, and reactive oxygen species (ROS) have important roles in the induction of oxidative stress, and thus, we used ELISA to detect their levels in IPEC-J2 cells. LPS and H_2_O_2_ (*p* < 0.05) stimulation significantly reduced SOD and CAT expression in IPEC-J2 cells, and this effect was reversed by baicalin treatment (Figures 4C,E). Meanwhile, LPS (*p* < 0.05) stimulation enhanced MDA and ROS levels, and these changes were similar reversed by baicalin exposure (Figures 4B,D).

### Effect of Baicalin on Nrf2 Signaling in IPEC-J2 Cells

**Figure 5:**
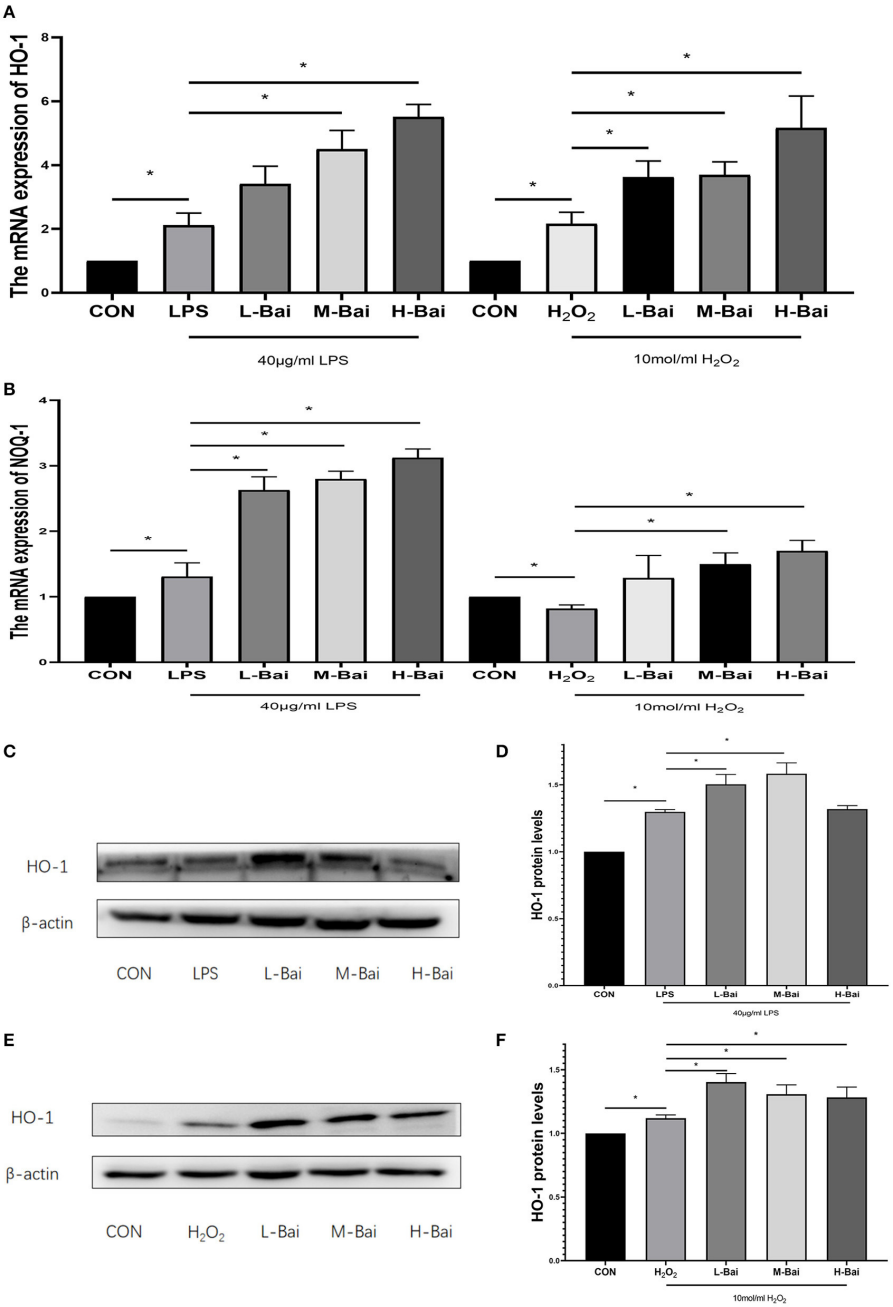
Effects of baicalin on key proteins and mRNAs in the Nrf2 signaling pathway in IPEC-J2 cells. Effect of baicalin on HO-1 mRNA expression in IPEC-J2 cells stimulated by LPS and H_2_O_2_ as determined by qPCR **(A)**. Effect of baicalin on NQO-1 mRNA expression in IPEC-J2 cells stimulated by LPS and H_2_O_2_ as determined by qPCR **(B)**. Immunofluorescence detection of nuclear Nrf2 protein expression in IPEC-J2 cells **(C)**. Western blotting was used to determine the expression of HO-1, Nrf2, β-actin, and lamin B **(D)**. HO-1 protein expression **(E)**.Cytoplasmic and nuclear Nrf2 protein expression **(F)**. Data are presented as the mean ± SEM (*n* = 3 per treatment, *p < 0.05 compared with the model group). CON, control; L-Bai, low-concentration baicalin; M-Bai, middle-concentration baicalin; H-Bai, high-concentration baicalin.

The signaling pathways for Nrf2 and heme oxygenase-1 (HO-1) are essential in oxidative stress. In IPEC-J2 cells, LPS and H_2_O_2_ treatment significantly elevated HO-1 mRNA expression (*p* < 0.05), which was reversed by baicalin in a concentration-dependent manner (*p* < 0.05, Figure 5A) Meanwhile H_2_O_2_, but not LPS, significantly increased quinone oxidoreductase-1 (NQO-1) mRNA expression in IPEC-J2 cells, whereas baicalin (*p* < 0.05) significantly increased NQO-1 mRNA expression at all examined concentrations (Figure 5B).

LPS and H_2_O_2_ (*p* < 0.05) stimulation significantly increased HO-1 protein expression, and its expression was further increased by pretreatment with low and middle concentrations of baicalin (*p* < 0.05, Figures 5C–F).

**Figure 6:**
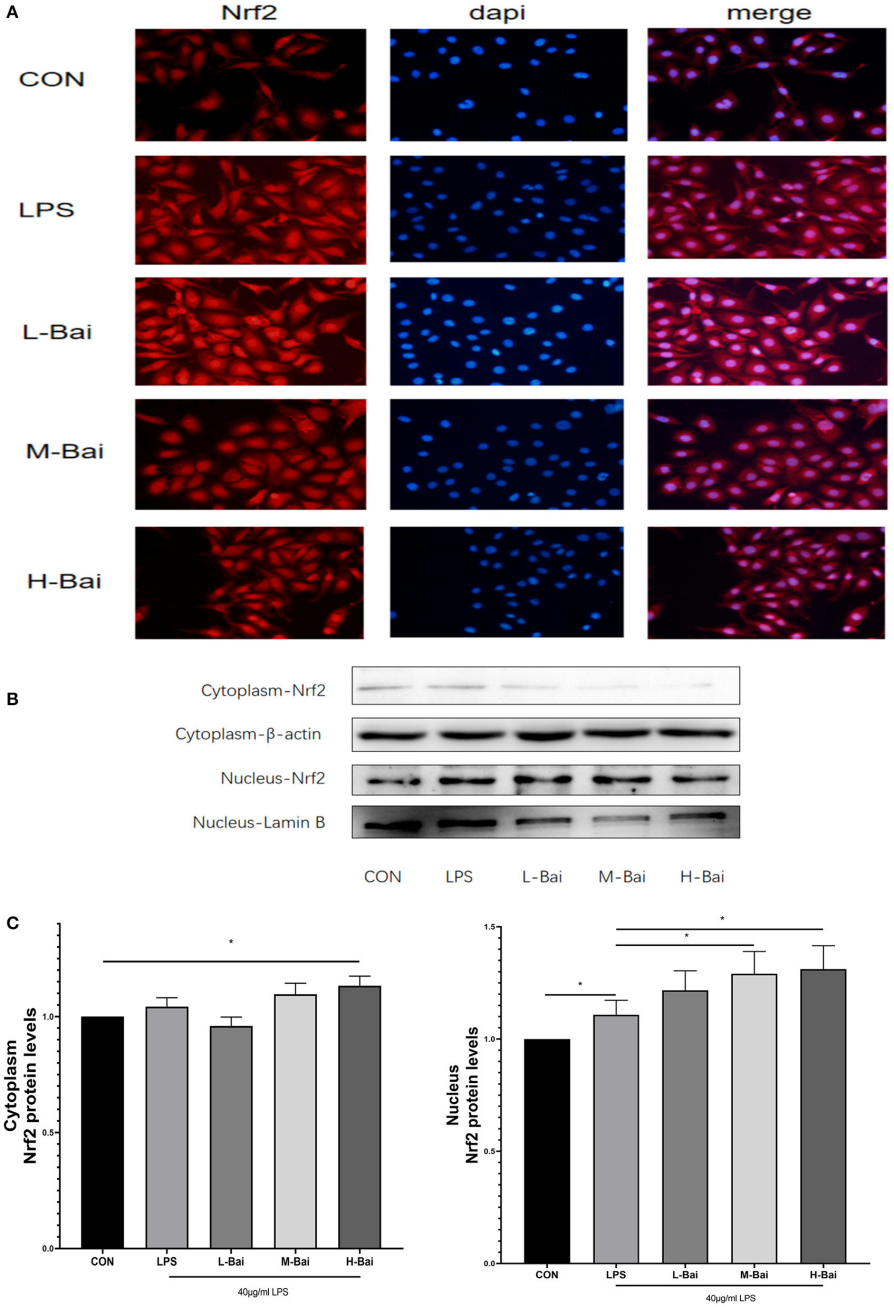
Effects of baicalin on key proteins in the Nrf2 signaling pathway in LPS-stimulated IPEC-J2 cells. Immunofluorescence detection of nuclear Nrf2 protein import in IPEC-J2 cells **(A)**. Western blotting was used to determine the expression of HO-1, Nrf2, β-actin, and lamin B **(B)**. Cytoplasmic and nuclear Nrf2 protein expression **(C)**. Data are presented as the mean ± SEM (*n* = 3 per treatment, *p < 0.05 compared with the model group). CON, control; L-Bai, low-concentration baicalin; M-Bai, middle-concentration baicalin; H-Bai, high-concentration baicalin.

According to fluorescence microscopy, LPS stimulation significantly increased Nrf2 protein accumulation in the nucleus in IPEC-J2 cells (Figure 6A), and further increases were observed in the presence of low and middle concentrations of baicalin. Meanwhile, high-concentration baicalin (*p* < 0.05) significantly increased Nrf2 protein expression in the cytoplasm of IPEC-J2 cells (Figures 6B,C). LPS (*p* < 0.05) stimulation significantly increased nuclear Nrf2 expression in IPEC-J2 cells, whereas middle- and high-concentration baicalin exposure further increased the nuclear accumulation of Nrf2 (Figures 6B,D).

**Figure 7:**
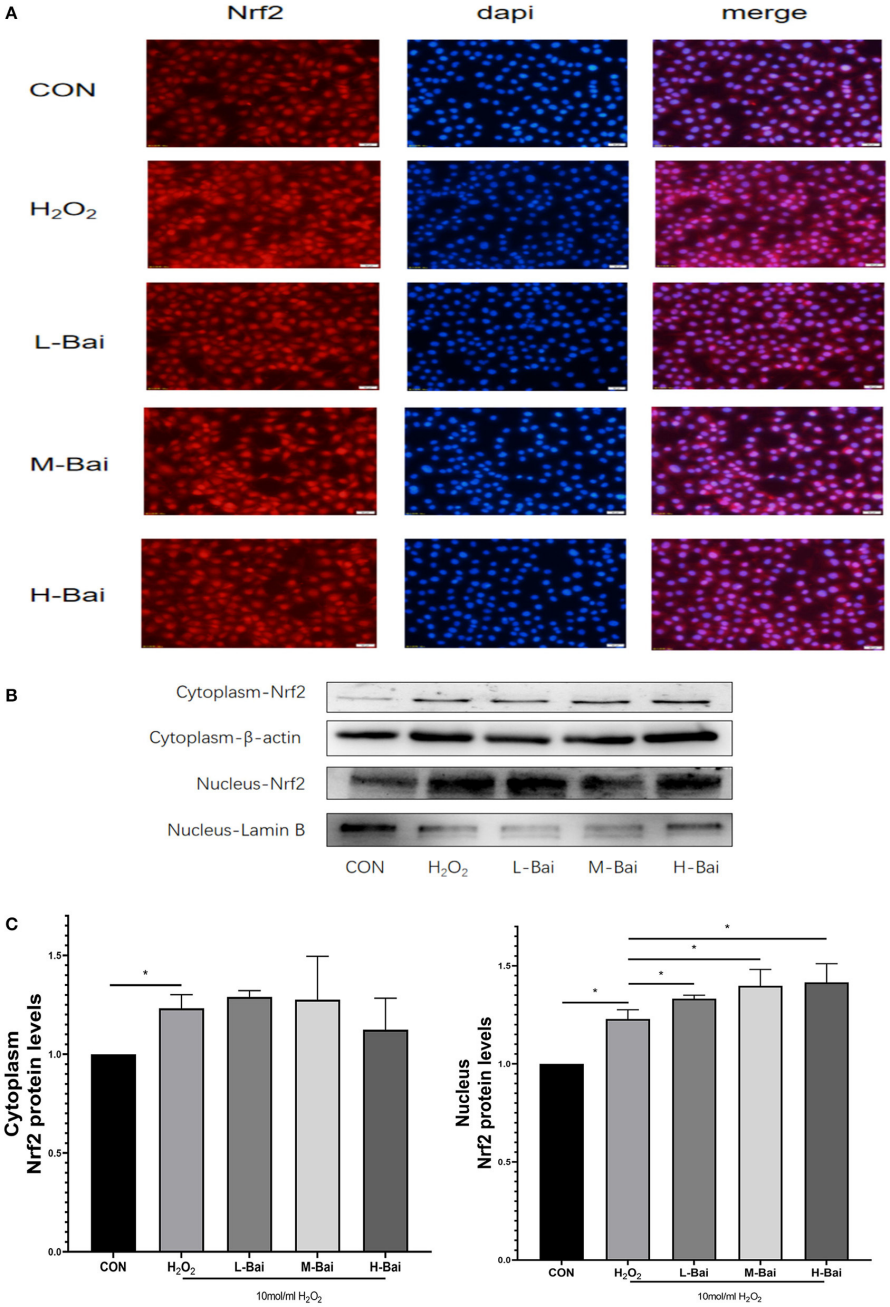
Effects of baicalin on key proteins in the Nrf2 signaling pathway in H_2_O_2_-stimulated IPEC-J2 cells. Immunofluorescence detection of nuclear Nrf2 protein import in IPEC-J2 cells **(A)**. Western blotting was used to determine the expression of HO-1, Nrf2, β-actin, and lamin B **(B)**. Cytoplasmic and nuclear Nrf2 protein expression **(C)**. Data are presented as the mean ± SEM (*n* = 3 per treatment, *p < 0.05 compared with the model group). CON, control; L-Bai, low-concentration baicalin; M-Bai, middle-concentration baicalin; H-Bai, high-concentration baicalin.

Similarly, H_2_O_2_ stimulation significantly increased Nrf2 protein accumulation in the nucleus in IPEC-J2 cells (Figure 7A), and this effect was potentiated by baicalin. H_2_O_2_ (*p* < 0.05) stimulation also significantly increased the cytoplasmic expression of Nrf2 (Figure 7C), but baicalin did not further increase Nrf2 protein expression in the cytoplasm.

## Discussion

Our result proved that baicalin might be used as a potential medicine for the treatment of piglets diarrhea by the way of anti-inflammatory and antioxidant. The concentration range of baicalin was selected by the viabilities of IPEC-J2 cells. Baicalin within the concentration 0–16 μg/mL had no obvious cytotoxicity to IPEC-J2 cells, but when the concentration of baicalin was greater than or equal to 32 μg/mL, it would significantly decrease the cell viabilities. Baicalin pretreatment enhanced viability, reduced inflammatory responses, and enhanced anti-oxidant enzyme activity in IPEC-J2 cells. Low-concentration baicalin had enhanced the viability of IPEC-J2 cells, which may be related to the biological function of baicalin in promoting cell proliferation (32). The viability of IPEC-J2 cells was considerably reduced by LPS and H_2_O_2_ stimulation, which was reversed by pretreatment with baicalin. So, pretreatment of IPEC-J2 cells with baicalin at concentrations less than 16 μg/mL can safely and effectively enhance cell viability. As a result, 4, 8 and 16 μg/mL of baicalin were determined as the low, medium and high doses respectively. TLRs are important receptors on the cell surface (33). TLR4 can recognize extracellular stimulation and then activate the NF-κB signaling pathway, which stimulates P-p65 protein accumulation in the nucleus and the subsequent induction of inflammatory responses, promoting the secretion of inflammatory factor such as TNF-α, IL-6, IL-1β and IL-8 (34). Moreover, IL-8 has the biological function of organizing pathogen invasion and eliminating pathogen as a chemokine (35). In this study, LPS stimulation significantly increased TLR4 mRNA expression in IPEC-J2 cells, similar to previous findings (36). Moreover, LPS stimulation significantly increased the protein and mRNA expression of pro-inflammatory factors (TNF-α, IL-6, IL-1β and IL-8) in IPEC-J2 cells (37), whereas pretreatment with baicalin concentration-dependently reversed this trend, indicating that baicalin did have capacity to suppress LPS-induced inflammation in IPEC-J2 cells. Baicalin can reduce LPS induced IL-8 secretion at mRNA and protein levels, suggesting that baicalin may play a key role in preventing pathogen invasion. Further, the protein expression of p65 and IκBα which were important components of the NF-κB signaling pathway were investigated (38, 39). LPS stimulation significantly increased P-p65 and P-IκBα protein expression, and phosphorylation of p65 increased its nuclear accumulation in IPEC-J2 cells. After entering the nucleus, P-P65 protein can induce the expression of the downstream inflammatory factors TNF-α, IL-6, IL-1β and IL-8 to initiate an inflammatory response (40). The results of immunofluorescence indicated that LPS stimulation significantly increased the nuclear entry of P-p65 in IPEC-J2 cells, whereas baicalin pretreatment exerted a concentration-dependent inhibitory effect on the nuclear entry of P-p65. It illustrated that baicalin alleviated the inflammatory responses of IPEC-J2 cells by inhibiting TLR4–NF-κB signaling pathway.

Oxidative stress can cause cells to undergo death and irreparable oxidative damage ROS (41, 42), which can cause oxidative stress and then trigger cell damage (43). It is generally believed that excessively ROS levels and the decreased antioxidant enzyme and propylene glycol (MDA) levels indicate the occurrence of oxidative stress. By contrast with the LPS-induced oxidative stress model, an oxidative stress model was created by treating IPEC-J2 cells with 10 mol/mL H_2_O_2_, as previously reported (44, 45). In the H_2_O_2_ -induced oxidative stress model, baicalin significantly increased the mRNA expression of HO-1 and NQO-1 and significantly increased nuclear protein expression of Nrf2, which revealed that baicalin could alleviate oxidative stress responses in IPEC-J2 cells. Previous studies revealed that LPS can excessive ROS production, which can cause oxidative stress and then trigger cell damage (46). Therefore, we detected the levels of ROS, MDA, and the anti-oxidant enzymes SOD and CAT in IPEC-J2 cells. LPS stimulation elevated MDA levels in IPEC-J2 cells, suggesting that oxidative stress occurs in this model. CAT and SOD play important roles in maintaining normal cellular redox balance (47). LPS stimulation significantly decreased CAT and SOD levels in IPEC-J2 cells, but pretreatment with baicalin decreased MDA levels and increased CAT and SOD levels. This indicates that baicalin can inhibit oxidative stress induced by LPS in IPEC-J2 cells. It can also be seen that baicalin ameliorates oxidative stress in the spleen of chickens during Mycoplasma gallisepticum infection (48). In the bovine endometrial epithelial cells, andrographolide could attenuates LPS-indcued inflammatory response via activating Nrf2 signaling pathway (49), while Nrf2 may also be an important target of baicalin. Baicalin-pretreated IPEC-J2 cells exhibited significantly increased HO-1 and NQO-1 mRNA expression, increased HO-1 protein expression, and significantly increased nuclear Nrf2 protein expression relative to the findings in LPS-treated IPEC-J2 cells. Baicalin activates the Nrf2 signaling pathway, which alleviates LPS-induced oxidative stress in IPEC-J2 cells. In this study, baicalin significantly inhibited inflammation and oxidative stress in IPEC-J2 cells. Notably, HO-1 protein expression was higher in the high-concentration baicalin group than in the middle-concentration group, which may be related to the negative feedback regulation in cells (50). High-concentration baicalin significantly increased the expression of anti-oxidant enzymes, which may reduce HO-1 protein expression. These results illustrated that baicalin could exert anti-oxidant activity in the oxidative stress model of IPEC-J2 cells by activating the Nrf2 signaling pathway.

**Figure 8:**
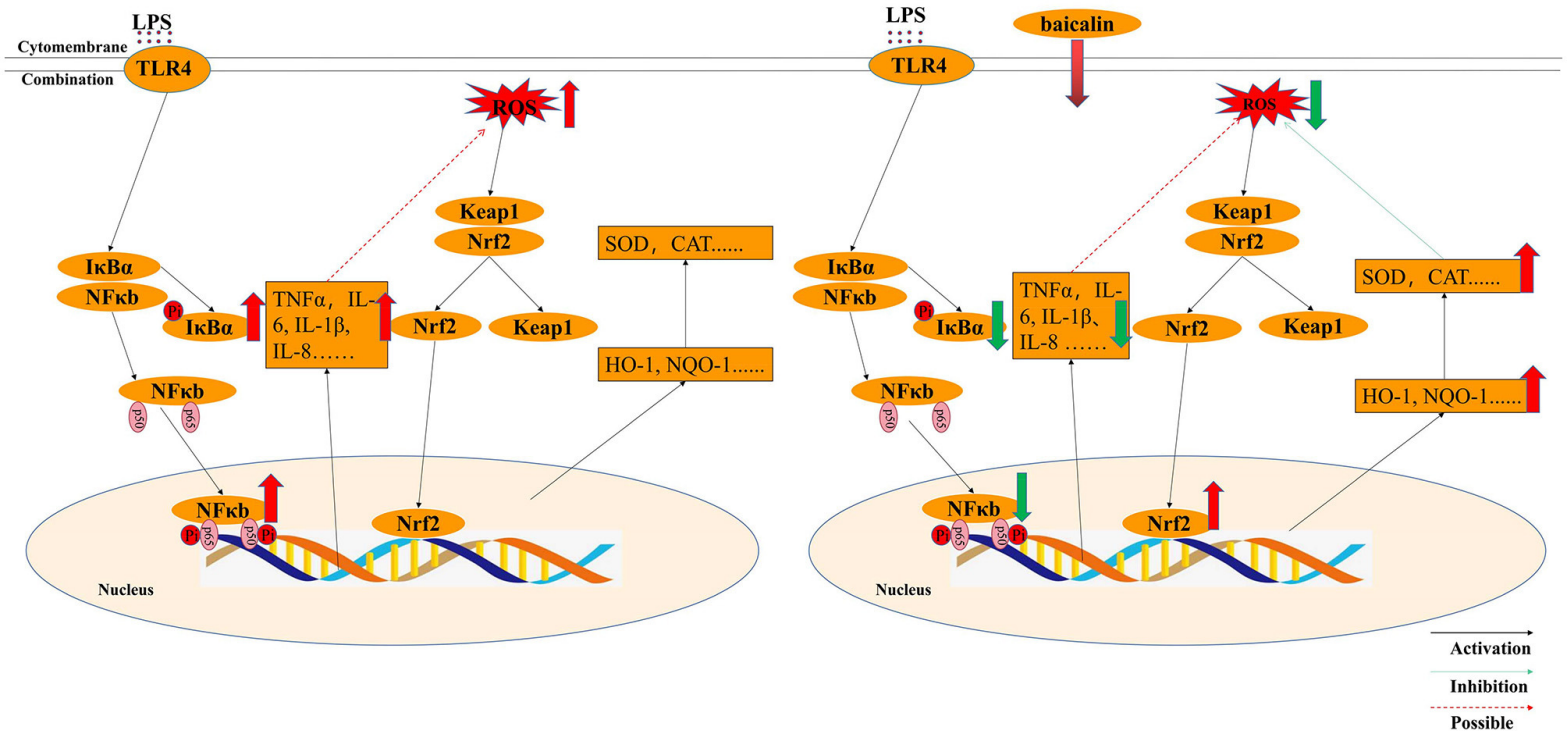
Molecular mechanism by which baicalin alleviated LPS-induced inflammatory responses and oxidative stress model in IPEC-J2 cells.

Although it is widely established that H_2_O_2_ can cause oxidative stress in cells by creating excessive ROS, but how LPS stimulation causes oxidative stress in the intestine of piglets is still unknown. It was believed that TNF-α can promote oxidative stress (25). In differentiated bone marrow-derived macrophages, TNF-α induced oxidative stress through the Keap1–Nrf2–TNF-α axis, which increased ROS accumulation (51). In Gpx4-deficient cells, inflammatory cytokines (TNF-α) could create a positive feedback loop involving ROS production, inflammation, and mutagenesis (52). In this study, LPS exposure enhance ROS levels and the expression of pro-inflammation cytokines such as TNF-α, IL-1β IL-6 and IL-8. Thus, it was supposed that inflammatory cytokines, especially TNF-α, might be the main factors of ROS production. LPS activated the NF-κB signaling pathway, increased the production of inflammatory factors and ROS, and decreased the expression of anti-oxidant enzymes, all of which promoted oxidative stress in IPEC-J2 cells. ROS content was similar in LPS- and H_2_O_2_-treated IPEC-J2 cells; however, ROS levels in baicalin-pretreated cells in the LPS-induced oxidative stress model were lower than that in the H_2_O_2_-induced oxidative stress model, which may be attributable to decreased cytokine expression and increased NQO-1 mRNA expression following baicalin exposure. In the process of LPS-induced oxidative stress, baicalin significantly suppressed LPS-induced cytokines by inhibiting the NF-κB signaling' pathway, activating the Nrf2 signaling' pathway, increasing the expression of anti-oxidant enzymes, and reducing the levels of ROS. Thus, baicalin can alleviate LPS-induced oxidative stress by inhibiting the NF-κB signaling pathway and stimulating the Nrf2–HO-1 signaling pathway in IPEC-J2 cells (Figure 8). In the process of LPS-induced oxidative stress, it seems that cytokines, especially TNF-α, seem to critical factors to crosstalk the inflammation response with oxidative stress, which need our deep research. Furthermore, we still want to explore how baicalin regulates the anti-inflammatory and antioxidant response in the intestine of piglets in vivo.

## Conclusion

In this paper, it was proved that LPS induced the process of oxidative stress via NF-κB and Nrf2–HO1 signaling pathways, which was familiar with the process of H_2_O_2_-induced oxidative stress. Baicalin can reduce inflammatory response by inhibiting NF-κB signal pathway, and it can reduce oxidative stress response by stimulating Nrf2-HO-1 signal pathway. All in all, pretreatment with baicalin can inhibite LPS-induced oxidative stress and protect the normal physiological function of IPEC-J2 cells via NF-κB and Nrf2–HO1 signaling pathways.

## Data Availability Statement

The original contributions presented in the study are included in the article/Supplementary Material, further inquiries can be directed to the corresponding author/s.

## Author Contributions

MB, XL, and SM: conceptualization, writing-review, and editing. MB, ML, XS, and XM: analysis. SC, JW, and ZY: data curation. MB: writing-original draft preparation. XL, YY, and XJ: supervision. XL and XJ: project administration, methodology, and funding acquisition. All authors contributed to the article and approved the submitted version.

## Funding

This work was supported by grants from the National Natural Science Foundation of China [No. 31902314], the Natural Science Foundation of Guangdong Province, China (No. 2019A1515011142), the Nanhai Scholars Program of Guangdong Ocean University (No. 002029002005) and the Program for Scientific Research Start-Up Funds of Guangdong Ocean University (101402/R17088), Shenzhen Projects for Basic Research [JCYJ20190813142005766] and Project of Enhancing School with Innovation of Guangdong Ocean University (GDOU230419057).

## Conflict of Interest

The authors declare that the research was conducted in the absence of any commercial or financial relationships that could be construed as a potential conflict of interest.

## Publisher's Note

All claims expressed in this article are solely those of the authors and do not necessarily represent those of their affiliated organizations, or those of the publisher, the editors and the reviewers. Any product that may be evaluated in this article, or claim that may be made by its manufacturer, is not guaranteed or endorsed by the publisher.
